# Effect of Oral Microbiota Composition on Metabolic Dysfunction-Associated Steatotic Liver Disease in the General Population

**DOI:** 10.3390/jcm14062013

**Published:** 2025-03-16

**Authors:** Satoshi Sato, Chikara Iino, Keisuke Furusawa, Kenta Yoshida, Daisuke Chinda, Kaori Sawada, Tatsuya Mikami, Shigeyuki Nakaji, Shinsaku Fukuda, Hirotake Sakuraba

**Affiliations:** 1 Department of Gastroenterology, Hematology, and Clinical Immunology, Hirosaki University Graduate School of Medicine, Hirosaki 036-8562, Japan; 2 Division of Endoscopy, Hirosaki University Graduate School of Medicine, Hirosaki 036-8562, Japan; 3 Department of Preemptive Medicine, Hirosaki University Graduate School of Medicine, Hirosaki 036-8562, Japan

**Keywords:** metabolic dysfunction-associated steatotic liver disease, oral microbiota, general population

## Abstract

**Background/Objective:** This study investigated the relationship between the composition of oral microbiota and metabolic dysfunction-associated steatotic liver disease (MASLD) in the general population. **Methods:** In total, 712 participants in a health check-up project were divided into four oral microbiota patterns by principal component analysis and cluster analysis; they were included in *Neisseria*, *Streptococcus*, *Fusobacterium*, and *Veillonella* groups. The *Neisseria* group had the largest number of patients and was used as a reference group to compare the incidence of MASLD and cardiometabolic criteria with the other groups. **Results:** In a multivariate analysis, the *Veillonella* group was a risk factor for MASLD independent of cardiometabolic criteria compared with the *Neisseria* group. The correlation between oral bacterial species and MASLD-related items showed that *Neisseria* was negatively correlated with controlled attenuation parameters, body mass index, waist circumference, hemoglobin A1c, alanine aminotransferase, and fatty liver index. *Veillonella* showed a positive correlation with controlled attenuation parameters, waist circumference, body mass index, blood pressure, triglycerides, alanine aminotransferase, aspartate aminotransferase, gamma-glutamyl transpeptidase, and fatty liver index, and a negative correlation with high-density lipoprotein cholesterol. In contrast, the *Streptococcus* and *Fusobacterium* groups were not clearly associated with MASLD. **Conclusions:** Maintaining oral hygiene and preventing periodontitis may contribute to preventing MASLD and extending a healthy lifespan.

## 1. Introduction

Fatty liver is asymptomatic; however, it can cause cardiovascular disease and hepatitis, and it is a risk factor for cirrhosis and liver cancer. Furthermore, recent studies have shown that fatty liver is not only associated with obesity but also with diabetes, dyslipidemia, hypertension, and atherosclerosis. Fatty liver is considered a hepatic phenotype of lifestyle-related diseases [1,2,3]. Fatty liver disease without drinking habits was previously called non-alcoholic fatty liver disease (NAFLD), but the name was changed to metabolic dysfunction-associated steatotic liver disease (MASLD) in 2023 [4]. With the name change from NAFLD to MASLD, the diagnostic criteria now specify that a patient must meet ≥1 of 5 cardiometabolic criteria (obesity, hypertension, diabetes, high-density lipoprotein [HDL] cholesterol, and high triglycerides), which is more closely related to lifestyle-related diseases. The link to lifestyle-related diseases has been deepened. Metabolic dysfunction-associated steatotic liver disease, a lifestyle-related disease, is on the increase worldwide, with a prevalence of 30% [5]. In contrast, periodontitis affects 20–50% of the world’s population and has been linked to diabetes and cardiovascular disease [6,7,8]. The oral environment is also involved in neurodegenerative diseases such as Parkinson’s disease [9]. Additionally, patients with MASLD have a higher risk of periodontitis [10]. Periodontitis is not only involved in the onset and progression of various lifestyle-related diseases but also a lifestyle-related disease that is associated with lifestyle habits, such as poor oral hygiene and smoking. Both MASLD and periodontal disease are lifestyle-related diseases that have been attracting attention in recent years, and prevention of both conditions is important in extending healthy life expectancy.

Numerous prior studies have demonstrated a significant association between the oral microbiota and MASLD [11]. Immunogenic factors, such as lipopolysaccharides and oral pathogenic bacteria from a periodontitis tissue, enter the liver hematogenously and contribute to the onset and progression of MASLD [12,13]. Additionally, host cells in the periodontal ligament triggered by the immune response to biofilm bacterium increase reactive oxygen species and inflammatory cytokines, such as tumor necrosis factor-alpha (TNF-α), IL-6, and interleukin (IL)-1β, which are related in MASLD development [14]. A mechanism has also been proposed in which MASLD develops as a result of dysbiosis caused by the migration of oral bacteria into the gut due to decreased gastric acid secretion. [11].

Although many previous studies have investigated the relationship between oral microbiota and fatty liver disease, there are still few epidemiological studies that have gone into a relationship with the cardiometabolic criteria following the change in the disease name from NAFLD to MASLD. Additionally, it is important to investigate the influence of oral microflora patterns on MASLD, in general, the population to develop preventive methods for MASLD, one of the lifestyle-related diseases.

Therefore, our study aimed to clarify the association between oral microflora patterns and MASLD in general residents.

## 2. Materials and Methods

### 2.1. Study Participants

**Figure 1:**
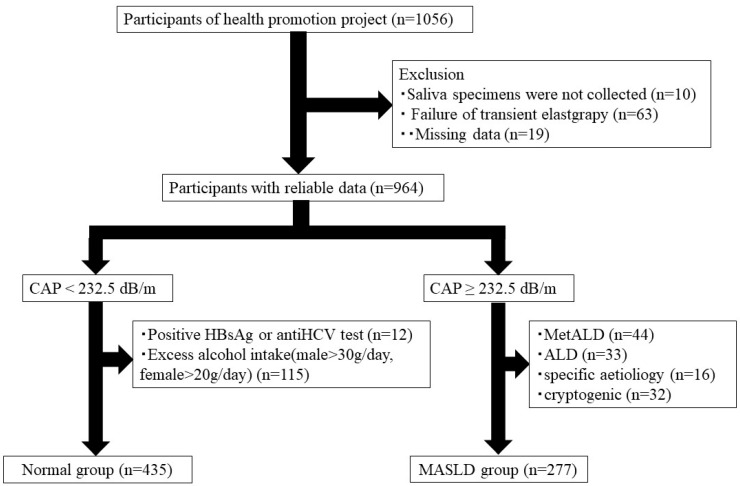
Flowchart of this study. CAP, controlled attenuation parameter; MASLD, metabolic dysfunction associated steatotic liver disease; ALD, alcohol-associated liver disease; HCV, hepatitis C virus.

Our study was conducted as one of the “Iwaki Health Promotion Project”, the community-based health promotion project targeting the general Japanese residents. The Iwaki Health Promotion Project is an ongoing community-based health promotion research program aimed at preventing lifestyle-related diseases and extending life expectancy among Japanese adults. The project targets inhabitants of the Iwaki district, Hirosaki City, and Aomori Prefecture, and was performed as a regular health check-up every year in June [15]. This project has been conducted annually since 2005, with around 1000 participants. All subjects participated voluntarily according to the public announcement, and various data, including physique, lifestyle data, medical history, microflora, and blood chemistry analysis data were collected from each participant. All participants were sufficiently informed of the purpose and procedures of our study and provided written consent. A total of 1056 adults (aged 19–88 years) who voluntarily responded to a public call participated in this study. The participants were excluded if they were unable to accurately assess fatty liver due to transient elastography failure or had missing values on any of the measures, and saliva specimens were not collected or missing data. Additionally, based on previous reports, steatotic liver disease (SLD) was identified with a cut-off value of 232.5 dB/m for the controlled attenuation parameter (CAP) value using FibroScan (Echosens, Paris, France) [16]. Approximately 435 participants were included in a normal group after excluding individuals with positive hepatitis B surface (HBs) antigen, positive anti-hepatitis C virus (HCV) antibody, or habitual drinkers (>30 g/day for men and 20 g/day for women) from the non-SLD group. In the SLD group, 277 participants who met the diagnostic criteria were included in the MASLD group [4]. We analyzed 712 patients (435 in the normal group and 277 in the MASLD group) (Figure 1).

### 2.2. Transient Elastography

The CAP and liver stiffness measurements were conducted by a FibroScan 530 (Echosens, Paris, France) using M and XL probes. All the tests were conducted by five professionally trained hepatologists. Measurements were excluded if the number of measurements was <10 or if the interquartile range ratio was ≥0.30; this is because they were unreliable. In a previous study, CAP values > 232.5 dB/m were defined as fatty liver [16].

### 2.3. Clinical Parameters

The following parameters were measured at the date of this project visit: age, sex, waist circumference, height, body mass index (BMI, calculated by dividing the weight [in kilograms] by the squared height [in meters]), anti-HCV test or HBsAg results, and levels of alanine aminotransferase, aspartate aminotransferase, gamma-glutamyl trans-peptidase, glucose, hemoglobin A1c (HbA1c), triglycerides, high-density HDL cholesterol, and low-density lipoprotein (LDL) cholesterol.

The fatty liver index was calculated as the following formula:

(e [0.953 × ln [triglycerides] + 0.139 × BMI + 0.718 × ln [GGT] + 0.053 × waist circumference − 15.745)/(1 + e [0.953 × ln [triglycerides] + 0.139 × BMI + 0.718 × ln [GGT] + 0.053 × waist circumference − 15.745]) × 100.

### 2.4. MASLD Diagnosis

Metabolic dysfunction-associated steatotic liver disease was diagnosed as fatty liver without drinking habits or other liver diseases, plus ≥1 of the following items: obesity, hyperglycemia, high blood pressure, high triglycerides, and reduced HDL cholesterol. Specific criteria included a waist circumference of ≥94 cm for males, and ≥80 cm for females or BMI of ≥23 kg/m^2^; fasting blood glucose of ≥100 mg/dL, postprandial blood glucose of ≥140 mg/dL, HbA1c of ≥5.7%, or treatment for type 2 diabetes mellitus; blood pressure ≥130/85 mmHg or antihypertensive treatment; triglycerides ≥150 mg/dL or treatment for dyslipidemia; and HDL cholesterol ≤40 mg/dL for males and ≤50 mg/dL for females [4].

### 2.5. Measurements of Oral Microbiota

The oral microbiota data were obtained using the following procedure: The participants were provided with a saliva sample kit beforehand, and saliva samples were collected on the day of the project at home. DNA was extracted from the bead-beaten saliva suspensions by an automated nucleic acid extraction system (Precision System Science, Chiba, Japan). The MagDEA DNA 200 (GC) reagent kit (Precision System Science, Chiba, Japan) was utilized for nucleic acid extraction. DNA extraction from saliva samples was completed within 4 months. Universal primer sets were utilized to amplify the V3-V4 regions of the 16S rRNA gene. The condition setting and solution preparation for PCR amplification were conducted as described previously [17]. PCR fragments purified utilizing PCR Cleanup Filter Plates (Merck Millipore, Burlington, MA, USA) were quantified by the real-time quantitative PCR. Purified PCR fragments were analyzed by paired-end sequencing of 2 × 300 cycles on a MiSeq™ system (Illumina, San Diego, CA, USA) to read DNA sequences. Paired-end reads were processed as follows: adapter sequences and low-quality bases (Q < 20) at the 3′ end of the reads were trimmed using Cutadapt (version: 1.13). The reads containing ambiguous bases N or shorter than 150 bp were excluded. The paired-end reads which met the criteria were merged into a single read called a “merged read”. Merged reads shorter than 370 bp or longer than 470 bp were excluded using the fastq_mergepairs subcommand in VSEARCH (version 2.4.3) [18]. Merged reads containing one or more identified sequencing errors were excluded. After eliminating the chimeric reads identified using the uchime_denovo subcommand of VSEARCH, the remaining merged reads were clustered with the minimum sequence similarity of 97% to obtain operational taxonomic units (OTUs). OUT taxonomic assignments were performed using the RDP classifier (commit hash: 701e229dde7cbe53d4261301e23459d91615999d) based on representative reads [19]. Predictions with a confidence score below 0.8 were treated as unclassified. The relative abundance of each bacterial genus in oral microbiota was calculated by dividing the read count of each bacterial genus by the total read count. In this study, 505 bacterial species were extracted.

### 2.6. Oral Microbiota Pattern Analysis

To assess the oral microbiota patterns, we performed principal component analysis (PCA) with varimax rotation on 47 oral bacteria species with a relative abundance of ≥1%. After that, the participants were classified into four oral microbiota patterns via PCA using non-hierarchal cluster analysis (k-means method). The effects of oral microbiota patterns on MASLD and related items were investigated.

### 2.7. Statistical Analysis

Continuous variables were described using medians and interquartile ranges. To compare the four groups, the Kruskal–Wallis test was employed, followed by Steel–Dwass multiple comparisons. Categorical variables were compared using the chi-square test with Bonferroni. The association between oral microbiota patterns and MASLD incidence was analyzed using univariate and multivariate analyses. Pearson’s correlation coefficient was used to examine the correlation between MASDL-related factors and oral microbiota species. Multiple regression models were used for the predictive analysis of MASLD-related factors and oral microbiota species. The models were adjusted for age, sex, smoking habits, and exercise habits. Prior to simple correlation and multiple regression analyses, all continuous parameters underwent log-transformed (natural logarithm) to achieve a closer approximation to a normal distribution.

All statistical analyses were conducted using the Statistical Package for the Social Sciences version 28.0 (SPSS Inc., Chicago, IL, USA) and R software (R Foundation for Statistical Computing, version R−4.1.1). A *p*-value of less than 0.05 was considered statistically significant.

### 2.8. Ethics Statement

Our study was conducted in accordance with the ethical standards of the Declaration of Helsinki and approved by the Ethics Committee of Hirosaki University School of Medicine (approval number and date: 2018–012, approved on 11 May 2018, and 2022–100, approved on 30 September). Informed consent was obtained from all the participants. All participants were informed of the purpose and procedures of the study, and they provided written consent.

## 3. Results

### 3.1. Participant Characteristics

Four components were extracted by PCA with varimax rotation (Table 1). Cluster analysis using the four factors obtained by PCA resulted in four groups. Each group was named based on the microbiota species that showed significantly higher relative abundance compared to the other groups, after comparing the oral microbiome patterns of each group.

The relative abundance of oral microbiota species among the four oral microbiota patterns is indicated in Table 2. The first group was named the *Neisseria* group, characterized by a relatively high abundance of *Neisseria* species. The second group, marked by a high relative abundance of *Streptococcus* species, was named the *Streptococcus* group. The third group was named the *Fusobacterium* group, due to its high relative abundance of *Fusobacterium* species. The fourth group was named the *Veillonella* group, based on its high relative abundance of *Veillonella* species. The *Neisseria* group had the largest number of participants and was used as the reference for comparison with the other three groups.

The *Fusobacterium* group had more males, higher BMI, waist circumference, fasting blood sugar, systolic blood pressure, triglycerides, alanine aminotransferase, gamma-glutamyl transpeptidase, CAP, and fatty liver index were higher than those in the *Neisseria* group. The prevalence of MASLD was 34.4% in the *Neisseria* group, 36.0% in the *Streptococcus* group, 45.5% in the *Fusobacterium* group, and 49.7% in the *Veillonella* group. Three or more of the five cardiometabolic criteria were possessed by 22.8% of the *Neisseria* group, 19.8% of the *Streptococcus* group, 35.6% of the *Fusobacterium* group, and 25.9% of the *Veillonella* group (Table 3).

**Figure 2:**
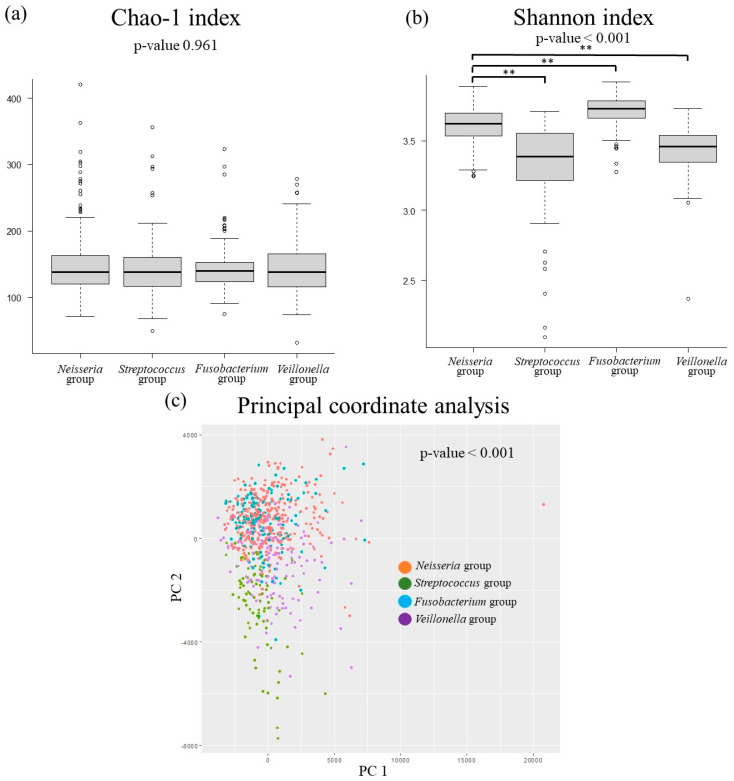
Comparative analyses of the diversity in oral microbiota. (**a**) Chao-1 index, (**b**) Shannon index, (**c**) principal coordinate analysis. ** *p* < 0.01.

Figure 2 shows the differences in diversity of the oral and gut microbiota. The Shannon index, an index of alpha diversity, was lower in the *Streptococcus* and *Veillonella* groups and higher in the *Fusobacterium* group than in the *Neisseria* group. The principal coordinate analysis, a measure of β-diversity, showed significant differences among the four oral bacterial patterns.

### 3.2. Risk Factors for Liver Fibrosis in Patients with MASLD

For the univariate analysis of risk factors with MASLD as the outcome, male, old age, smoking habit, obesity/central obesity, hyperglycemia or diabetes, high blood pressure, high triglycerides, and reduced HDL-cholesterol were significant risk factors for MASLD. Additionally, the *Neisseria*, *Fusobacterium*, and *Veillonella* groups were high-risk factors for MASLD. In the multivariate analysis, obesity/central obesity, hyperglycemia or diabetes, high triglycerides, reduced HDL-cholesterol, and the *Veillonera* group were risk factors for MASLD (Table 4).

### 3.3. The Relationship Between MASLD-Related Items and Oral Microbiota

Table 5 presents a summary of the single correlation analyses between MASLD-related items and the oral microbiota. *Neisseria* negatively correlated with CAP level, BMI, waist circumference, blood glucose, HbA1c, alanine aminotransferase, gamma-glutamyl transpeptidase, and fatty liver index, positively correlated with HDL cholesterol. In contrast, *Veillonella* showed a positive correlation with CAP level, BMI, waist circumference, blood pressure, blood glucose, HbA1c, aspartate aminotransferase, and alanine aminotransferase, gamma-glutamyl transpeptidase, and a negative correlation with HDL cholesterol.

Subsequently, we performed a multiple regression analysis, where the dependent variables were MASLD-related items, and the independent variables were sex, age, smoking, and exercise habits in addition to the oral microbiota (Table 6). *Neisseria* showed negative correlations with CAP, BMI, waist circumference, HbA1c, alanine aminotransferase, and fatty liver index. *Streptococcus* showed negative correlations with CAP level, waist circumference, triglycerides, alanine aminotransferase, gamma-glutamyl transpeptidase, and fatty liver index. *Fusobacterium* showed a positive correlation with alanine aminotransferase and gamma-glutamyl transpeptidase. In contrast, *Veillonella* showed the same correlation as a single correlation except for blood glucose and HbA1c. On the other hand, both standardized coefficient and coefficient of determination were low.

## 4. Discussion

This study was conducted to epidemiologically investigate the influence of oral microbiota patterns on MASLD in the general population. The results showed that the *Veillonella* group had a higher incidence of MASLD than the *Neisseria* group. Furthermore, in a multivariate analysis with cardiometabolic criteria as independent variables, the *Veillonella* group was also identified as an independent risk factor for MASLD. Additionally, this study found that oral *Veillonella* species were associated with increased liver fat content and worsening cardiometabolic criteria, while *Neisseria* species were associated with improvements in these parameters. Disorders of oral microflora such as periodontal disease are associated with various systemic diseases via periodontal disease-causing bacteria and inflammatory cytokines such as TNFα [6,20,21]. For example, aspiration pneumonia caused by periodontal disease-causing bacteria is a major cause of death in elderly people [22,23,24]. In patients with periodontal disease, insulin resistance and atherosclerosis are induced, leading to diabetes and cerebrovascular disease [7,8]. It has also been reported that periodontal disease is involved in osteoporosis and neurodegenerative diseases such as Parkinson’s disease [9,25]. The association between oral bacteria and liver disease has long been pointed out [26,27]. Periodontal disease is considered a risk factor for non-alcoholic fatty liver disease, and research is being conducted to determine whether it can lead to the prevention and treatment of MASLD/MASH, for which a treatment method has not yet been established [10,11,13].

In this study, the oral microbiota patterns were divided into four groups by PCA and cluster analysis, and the group with the largest number of people was the *Neisseria*-rich group. *Neisseria* and *Veillonella* are the major commensal bacteria in the oral cavity, but while *Neisseria* contributes to a healthy periodontal condition, *Veillonella* is known to contribute to poor health, including involvement in obesity, aging, and periodontal disease [28]. In previous studies conducted on Japanese individuals, the oral microbiota was divided into three groups: *Prevotella/Veillonella*, *Streptococcus*, and *Porphyromonas*/*Neisseria/Haemophilus*/*Aggregatibacter* groups. The *Prevotella/Veillonella* group reported worse periodontal disease [29]. This grouping is generally consistent with our study; therefore, the oral microbiota patterns of our study participants appear to be that of the general Japanese pattern.

The diversity of the four oral microbiota patterns in this study was different. In the gut microbiota, higher diversity has been reported to be healthier, while lower diversity has been associated with poor health [30]. However, with respect to oral microbiota, diversity is considered to be rich in an unhygienic oral environment [31]. In this study, the *Veillonella* group, which was a risk factor for MASLD, had a low diversity of oral bacteria, which was different from previous studies. However, the results of the relationship between oral bacterial diversity and fatty liver disease differ according to diversity indices (Chao-1 index, Shannon index, etc.), and a certain consensus has not been reached [32,33].

In this study, the *Neisseia*-rich group was found to be a lower risk factor for MASLD than the other groups. The genus *Neisseia* is one of the most abundant taxa of Gram-negative bacteria in the oral cavity [34]. A high abundance of *Neisseria* in the oral cavity is associated with oral health [35,36]. In previous studies investigating the relationship between MAFLD and the oral microbiota, it was reported that patients with MAFLD had decreased oral *Neisseria* [32,33]. In this study, oral *Neisseria* was negatively correlated with fasting blood glucose and HbA1c. However, the prevalence of oral *Neisseria* is elevated in diabetic individuals, and the underlying mechanism is hypothesized to involve impaired nitric oxide bioavailability, resulting in exacerbated insulin resistance [37,38]. Although the previous study compared diabetes with healthy individuals, the participants in this study compared fatty liver disease with healthy individuals. This difference in survey participants may be a reason for the different results from the previous study. Numerous studies reported that *Neisseria* contributes to oral health, and is decreased in fatty liver disease, suggesting that it may act in the direction of improving blood glucose for MASLD. However, the mechanism by which oral *Neisseria* exerts a healthy effect on the body has not yet been elucidated and is an issue for further investigation.

*Veillonella* is a species of Gram-negative anaerobic bacteria that primarily inhabits the oral cavity. The presence of *Veillonella* in the oral cavity is associated with increased production of inflammatory cytokines and periodontal infection [28,39,40]. *Veillonella* species are the major oral bacteria, but they are also present in the gut and are known to be increased in NAFLD and cirrhosis [41,42]. In addition to CAP level and fatty liver index, this study found that oral *Veillonella* was positively correlated with BMI, waist circumference, blood pressure, triglycerides, and hepatic LDL cholesterol, and negatively correlated with HDL cholesterol. Previous studies have reported that *Veillonella* migrating from the oral cavity to the intestine aggravates obesity and hypertension [43,44,45]. This study suggests that increased oral *Veillonella* may be involved in the development and progression of MASLD via obesity, lipid metabolism, and elevated blood pressure. The *Veillonella* group was also found to be a risk factor for MASLD in a multivariate analysis with cardiometabolic criteria as independent variables in this study. Oral *Veillonella* correlates with increased production of inflammatory cytokines [39,40]. Periodontitis has been shown to contribute to the development and progression of NAFLD through the mediation of inflammatory cytokines, including interleukin (IL)-1β, IL-6, and TNFα [13]. Our findings suggest that the proliferation of oral *Veillonella* may infect the pathogenesis of MASLD via an inflammatory cytokine-mediated pathway, independent of obesity, or metabolic disorders.

The *Fusobacterium* and *Streptococcus* groups were not significantly associated with MASLD in our study. In particular, the *Fusobacterium* group was not found to be a significant risk factor for MASLD in multivariate analysis despite having a higher BMI, blood pressure, and triglycerides than the other groups. Gut *Fusobacterium* is also increased in patients with NAFLD/NASH [46,47]. The fact that *Fusobacterium necrophorum*, one of the causative agents of periodontal disease, is often detected in liver abscesses suggests a strong relationship between the oral cavity and the liver [48]. On the other hand, in this study, *Neissria* and *Veillonella* showed a significant correlation with CAP values, but Fusobacterium showed no correlation with CAP values. Furthermore, the *Fusobacterium* group had a higher prevalence of cardiometabolic criteria than the other groups, but the prevalence of MASLD was not significantly higher. Although the detailed mechanism is unclear, it is possible that Fusobacterium has a relatively low direct effect on liver fat mass compared to *Neisseria* and *Veillonella*, and thus was not found to be a significant risk factor for MASLD in the multivariate analysis.

Gut *Streptococcus* has been reported to be increased in patients with NAFLD [49]. Oral *Streptococcus* is also increased in patients with fatty liver disease, and oral *Streptococcus* correlates with obesity and insulin resistance [33,50]. The previous study included patients with NAFLD/NASH having advanced fibrosis, while this study included the general population health check-up recipients. Furthermore, in this study, the CAP value of 232.5 dB/m on the Fibroscan was used as the cut-off value for fatty liver, which is a loose value that includes mild fatty liver, as fat is histologically accumulated in liver cells by >5% [16]. Our findings indicate that *Streptococcus* and *Fusobacterium* may play a less significant role in the pathogenesis and progression of MASLD among individuals with mild fatty liver.

Several limitations should be acknowledged in this study. Firstly, the study population was geographically restricted to a single region within Japan, which may limit the generalizability of our findings to other ethnic populations. Second, the number of remaining teeth and oral hygiene status, such as the presence of periodontal disease, have not been adequately assessed. By assessing oral hygiene, it may be possible to advocate for methods of prevention and treatment of MASLD through an oral approach. Thirdly, the diagnosis of fatty liver was conducted using FibroScan instead of liver biopsy. Invasive liver biopsy is not appropriate as part of health checkups in the general population and was not feasible in this study. Fourth, some oral bacterial species were significantly correlated with MASLD-related items, but the standardized coefficient and coefficient of determination were low. The effect of oral bacteria on MASLD is influenced by a variety of confounding factors, suggesting that the association is weak. Fifth, this study could not clearly distinguish whether the differences in oral microbiota were causal or consequential. Future bioinformatic analysis may lead to further clarification of the pathophysiology. The above limitation should be considered in interpreting the results of this study.

## 5. Conclusions

Our study revealed that variations in oral microbial composition were associated with the onset and progression of MASLD in the general population. The oral bacterial pattern identified as a risk factor for MASLD in this study was rich in *Veillonella*, which causes periodontitis. Additionally, oral *Veillonella* acted in the direction of worsening cardiometabolic criteria and liver fat content. Maintaining oral hygiene and preventing periodontitis may contribute to preventing MASLD and extending a healthy lifespan.

## Figures and Tables

**Table 1:** Factor loading matrix for oral microbiota patterns identified by the principal component analysis.

Classification	Species	Factor 1	Factor 2	Factor 3	Factor 4
Phylum	*Actinobacteria*	0.01	0.129	−0.092	0.029
	*Bacteroidetes*	0.005	−0.111	−0.002	−0.028
	*Candidatus Saccharibacteria*	−0.044	−0.001	−0.106	−0.004
	*Firmicutes*	0.079	0.017	0.083	−0.015
	*Fusobacteria*	0.035	0.039	0.03	0.164
	*Proteobacteria*	−0.064	−0.021	0.037	−0.017
Class	*Actinobacteria*	0.01	0.129	−0.092	0.029
	*Bacilli*	0.035	0.028	0.066	−0.04
	*Bacteroidia*	0.008	−0.111	−0.004	−0.033
	*Clostridia*	0.029	0.023	−0.029	0.116
	*Fusobacteriia*	0.035	0.039	0.03	0.164
	*Gammaproteobacteria*	0.028	−0.009	0.124	0.036
	*Negativicutes*	0.104	−0.034	0.062	0.016
Order	*Actinomycetales*	−0.005	0.143	−0.089	0.035
	*Bacteroidales*	0.008	−0.111	−0.004	−0.033
	*Betaproteobacteria*	−0.102	−0.021	−0.027	−0.047
	*Clostridiales*	0.03	0.023	−0.029	0.117
	*Coriobacteriales*	0.043	−0.013	−0.031	−0.011
	*Fusobacteriales*	0.035	0.039	0.03	0.164
	*Lactobacillales*	0.036	0.025	0.065	−0.043
	*Neisseriales*	−0.102	−0.021	−0.028	−0.047
	*Pasteurellales*	0.029	−0.01	0.124	0.036
	*Selenomonadales*	0.104	−0.034	0.062	0.016
Family	*Actinomycetaceae*	0.013	0.066	−0.098	0.051
	*Carnobacteriaceae*	−0.018	0.032	0.026	−0.007
	*Coriobacteriaceae*	0.043	−0.013	−0.031	−0.011
	*Fusobacteriaceae*	0.026	0.027	0.053	0.143
	*Lachnospiraceae*	0.04	0.023	−0.036	0.094
	*Micrococcaceae*	−0.012	0.111	−0.033	0.006
	*Neisseriaceae*	−0.102	−0.021	−0.028	−0.047
	*Pasteurellaceae*	0.029	−0.01	0.124	0.036
	*Porphyromonadaceae*	−0.044	−0.008	0.016	0.026
	*Prevotellaceae*	0.021	−0.104	−0.009	−0.039
	*Streptococcaceae*	0.041	0.022	0.066	−0.043
	*Veillonellaceae*	0.104	−0.034	0.062	0.016
Genus	*Actinomyces*	0.012	0.066	−0.098	0.051
	*Atopobium*	0.042	−0.015	−0.032	−0.013
	*Fusobacterium*	0.026	0.027	0.053	0.143
	*Granulicatella*	−0.018	0.032	0.026	−0.007
	*Haemophilus*	0.029	−0.01	0.124	0.037
	*Neisseria*	−0.102	−0.021	−0.028	−0.047
	*Porphyromonas*	−0.045	−0.01	0.016	0.023
	*Prevotella*	0.027	−0.098	−0.009	−0.046
	*Rothia*	−0.012	0.111	−0.033	0.006
	*Saccharibacteria_genera_incertae_sedis*	−0.044	−0.001	−0.106	−0.004
	*Streptococcus*	0.041	0.022	0.066	−0.043
	*Veillonella*	0.105	−0.035	0.069	0.013

**Table 2:** Relative abundance of oral microbiota genera species among four oral microbiota patterns.

Genera	First Group n = 334	Second Group n = 86	Third Group n = 149	Fourth Group n = 143
*Actinomyces*	6.4 (4.5–8.9)	7.1 (3.3–11.2)	6.9 (4.6–10.3)	6.1 (4.5–7.3)
*Atopobium*	1.5 (0.7–2.8)	1.4 (0.5–2.5)	1.5 (0.6–2.7)	2.5 (0.9–4.0)
*Fusobacterium*	2.0 (1.2–2.7)	0.9 (0.4–1.8)	4.1 (2.8–5.6)	1.3 (0.5–2.2)
*Granulicatella*	1.2 (0.8–1.8)	1.9 (1.2–2.5)	1.2 (0.7–1.7)	1.2 (0.7–1.9)
*Haemophilus*	4.2 (2.1–6.1)	4.3 (1.2–7.6)	4.4 (2.3–7.6)	4.3 (1.9–8.0)
*Neisseria*	10.6 (4.2–17.9)	2.3 (0.4–5.4)	6.9 (2.6–11.1)	1.6 (0.5–4.8)
*Porphyromonas*	1.8 (0.6–4.4)	0.3 (0.1–1.1)	2.8 (1.3–5.1)	0.5 (0.2–1.5)
*Prevotella*	16.8 (10.6–21.9)	7.0 (3.3–11.1)	14.5 (8.7–20.6)	18.6 (11.8–23.8)
*Rothia*	3.1 (1.8–5.3)	13.7 (9.2–20.3)	2.8 (1.5–5.2)	4.8 (2.9–6.3)
*Saccharibacteria_genera_incertae_sedis*	9.0 (4.8–14.5)	2.3 (0.5–4.5)	6.6 (3.4–11.2)	2.6 (0.9–5.6)
*Streptococcus*	17.3 (13.8–21.1)	32.7 (25.5–38.9)	15.5 (11.9–19.6)	26.2 (22.0–32.1)
*Veillonella*	7.4 (5.3–9.6)	8.3 (5.7–20.3)	8.3 (6.4–10.6)	12.7 (9.8–15.7)

Data are presented as median (range).

**Table 3:** Participants’ characteristics among the oral microbiota patterns.

	*Neisseria* Group n = 334	*Streptococcus* Group n = 86	*Fusobacterium* Group n = 149	*Veillonella* Group n = 143	*Neisseria* vs. *Streptococcus*	*Neisseria* vs. *Fusobacterium*	*Neisseria* vs. *Veillonella*
sex, male	94 (28.1%)	41 (47.7%)	62 (41.6%)	40 (28.0%)	0.005	0.029	0.999
Age (year)	50.0 (37.0–64.0)	57.0 (38.0–68.0)	57.0 (42.0–66.0)	56.0 (39.0–66.0)	0.225	0.114	0.183
BMI (kg/m^2^)	22.2 (19.6–24.6)	21.8 (20.2–23.6)	23.2 (20.7–25.8)	22.3 (20.0–25.1)	0.964	0.009	0.639
Waist circumference (cm)	74.0 (67.2–82.5)	74.2 (68.9–82.8)	79.0 (71.0–86.4)	74.4 (68.0–83.8)	0.762	<0.001	0.667
Fasting blood sugar (mmHg)	90.0 (85.0–98.0)	92.5 (86.8–100.3)	92.0 (87.5–100.5)	92.0 (85.0–98.0)	0.263	0.041	0.794
HbA1c (%)	5.7 (5.5–5.9)	5.7 (5.5–6.0)	5.7 (5.5–5.9)	5.7 (5.5–5.9)	0.633	0.890	0.895
Systolic blood pressure (mmHg)	120.0 (109.0–131.3)	121.5 (110.5–133.5)	127.0 (114.5–139.0)	123.0 (111.0–134.0)	0.997	0.004	0.498
Diastolic blood pressure (mmHg)	76.0 (69.0–83.0)	76.0 (69.0–82.8)	78.0 (69.5–87.0)	77.0 (71.0–86.0)	0.987	0.188	0.196
Triglycerides (mg/dL)	72.0 (50.0–105.0)	77.0 (53.8–97.3)	82.0 (57.0–123.5)	75.0 (54.0–110.0)	0.962	0.036	0.663
HDL cholesterol (mg/dL)	63.0 (53.0–74.3)	62.0 (54.8–78.3)	62.0 (50.0–75.0)	64.0 (55.0–78.0)	0.907	0.999	0.475
LDL cholesterol (mg/dL)	116.0 (96.8–135.3)	112.0 (98.5–136.5)	119.0 (99.5–142.0)	118.0 (99.0–138.0)	0.999	0.299	0.535
Aspartate aminotransferase (IU/L)	20.0 (17.0–24.0)	22.0 (17.0–25.0)	21.0 (17.5–26.0)	20.0 (17.0–25.0)	0.447	0.099	0.955
Alanine aminotransferase (IU/L)	17.0 (12.0–23.0)	18.0 (13.0–23.0)	20.0 (14.0–29.0)	17.0 (13.0–24.0)	0.762	0.006	0.646
γ-Glutamyl TransPeptidase (IU/L)	19.0 (14.0–31.0)	21.0 (15.0–35.0)	21.0 (17.0–35.0)	20.0 (15.0–30.0)	0.355	0.017	0.739
CAP (dB/m)	208.0 (168.8–251.3)	211.5 (174.5–261.3)	223.0 (195.0–274.0)	228.0 (185.0–267.0)	0.943	0.002	0.084
LSM (kPa)	4.3 (3.5–5.4)	4.1 (3.5–5.6)	4.4 (3.6–5.3)	4.3 (3.6–5.4)	0.982	0.986	0.999
Fatty liver index	10.7 (4.1–28.4)	11.8 (4.7–23.0)	20.2 (6.8–45.8)	13.4 (5.7–31.7)	0.868	<0.001	0.338
Smoking habit	35 (10.5%)	17 (19.8%)	19 (12.8%)	22 (15.4%)	0.190	0.999	0.999
Exercise habit	57 (17.1%)	18 (20.9%)	30 (20.1%)	18 (12.6%)	0.999	0.999	0.999
MASLD	115 (34.4%)	31 (36.0%)	66 (44.3%)	65 (45.5%)	0.999	0.300	0.180
Cardiometabolic risk factors							
High blood pressure	144 (43.1%)	44 (51.2%)	91 (61.1%)	71 (49.7%)	0.999	0.002	0.999
Obesity/central obesity	85 (25.4%)	19 (22.1%)	58 (38.9%)	40 (28.0%)	0.999	0.023	0.999
Hyperglycemia or diabetes	182 (54.5%)	56 (65.1%)	86 (57.7%)	84 (58.7%)	0.590	0.999	0.999
Resuce HDL-cholesterol	12.3%	8 (9.3%)	19 (12.8%)	11 (7.7%)	0.999	0.999	0.999
High triglycerides	68 (20.4%)	17 (19.8%)	41 (27.5%)	30 (21.0%)	0.999	0.630	0.999
Cardiometabolic crieria ≥ 3	76 (22.8%)	17 (19.8%)	53 (35.6%)	37 (25.9%)	0.999	0.028	0.999

Data are presented as numbers (%) or median (range). HbA1c, hemoglobin A1c; BMI, body mass index; HDL, high-density lipoprotein; LDL, low density; CAP, controlled attenuation parameter; LSM, liver stiffness measure; MASLD, metabolic dysfunction-associated steatotic liver disease.

**Table 4:** The univariable and multivariate analyses of risk factors for MASLD.

	Univariable				Multivariable			
	OR	95%CI		*p*-Value	OR	95%CI		*p*-Value
Male	1.64	1.19	2.25	0.002	1.41	0.95	2.09	0.084
Age	1.02	1.01	1.03	<0.001	1.01	0.99	1.02	0.405
smoking habit	1.56	1.01	2.42	0.045	1.51	0.87	2.61	0.140
exercise habit	1.14	0.77	1.69	0.522	0.93	0.58	1.49	0.773
Obesity/central obesity	7.28	5.06	10.50	<0.001	4.91	3.27	7.35	<0.001
Hyperglycemia or diabetes	4.22	3.01	5.92	<0.001	2.90	1.92	4.36	<0.001
High blood pressure	2.55	1.87	3.48	<0.001	1.42	0.95	2.14	0.092
High triglycerides	3.69	2.54	5.35	<0.001	2.29	1.48	2.55	<0.001
Reduce HDL-cholesterol	4.54	2.72	7.58	<0.001	2.70	1.47	4.95	<0.001
Oral microbiota pattern								
*Neisseria* group	1.00				1.00			
*Streptococcus* group	1.07	0.65	1.76	0.779	0.96	0.54	1.72	0.894
*Fusobacterium* group	1.51	1.02	2.25	0.039	1.08	0.67	1.73	0.767
*Veillonella* group	1.59	1.06	2.37	0.023	1.68	1.05	2.70	0.031

OR, odds ratio; CI, confidence interval; MASLD, metabolic dysfunction-associated steatotic liver disease.

**Table 5:** Single correlation analyses between MASLD-related items and oral microbiota.

	*Neisseria*		*Streptococcus*		*Fusobacterium*		*Veillonella*	
	r	*p*-Value	r	*p*-Value	r	*p*-Value	r	*p*-Value
CAP	−0.132	<0.001	−0.094	0.012	0.035	0.345	0.158	<0.001
BMI	−0.121	0.001	−0.054	0.153	0.014	0.712	0.138	<0.001
Waist circumference	−0.124	0.001	−0.048	0.203	0.045	0.236	0.118	0.002
Systolic blood pressure	−0.065	0.084	−0.049	0.192	0.001	0.988	0.112	0.003
Diastolic blood pressure	−0.067	0.072	0.007	0.846	−0.028	0.462	0.097	0.010
Blood glucose	−0.092	0.014	0.013	0.725	−0.033	0.382	0.095	0.012
HbA1c	−0.109	0.004	0.027	0.465	−0.075	0.046	0.104	0.006
Triglycerides	−0.124	0.001	−0.05	0.186	−0.001	0.970	0.148	<0.001
HDL cholesterol	0.076	0.041	0.023	0.533	0.015	0.689	−0.079	0.034
Aspartate aminotransferase	−0.06	0.107	−0.053	0.155	0.072	0.054	0.092	0.014
Alanine aminotransferase	−0.107	0.004	−0.078	0.037	0.115	0.002	0.089	0.018
Gamma-glutamyl transpeptidase	−0.14	<0.001	−0.075	0.046	0.042	0.267	0.160	<0.001
Fatty liver index	−0.083	0.026	−0.063	0.092	0.095	0.011	0.070	0.062
LSM	−0.04	0.283	0.018	0.637	0.030	0.425	0.020	0.602

r, Pearson’s correlation coefficient; CAP, controlled attenuation parameter; BMI, body mass index; HbA1c, hemoglobin A1c; HDL, high-density lipoprotein; LSM, liver stiffness measure; MASLD, metabolic dysfunction-associated steatotic liver disease.

**Table 6:** Multiple analyses between MASLD-related items and oral microbiota.

	*Neisseria*			*Streptococcus*			*Fusobacterium*			*Veillonella*		
	β	*p*	R^2^	β	*p*	R^2^	β	*p*	R^2^	β	*p*	R^2^
CAP	−0.092	0.017	0.065	−0.125	0.001	0.030	0.053	0.178	0.028	0.137	<0.001	0.038
Body mass index	−0.099	0.010	0.067	−0.071	0.070	0.021	0.001	0.972	0.025	0.137	<0.001	0.038
Waist circumference	−0.097	0.023	0.065	−0.090	0.040	0.022	0.030	0.495	0.026	0.133	0.002	0.034
Systolic blood pressure	−0.058	0.150	0.061	−0.059	0.155	0.019	−0.002	0.969	0.025	0.101	0.015	0.029
Diastolic blood pressure	−0.057	0.136	0.061	0.007	0.855	0.016	−0.040	0.306	0.027	0.088	0.023	0.028
Blood glucose	−0.059	0.135	0.061	0.001	0.981	0.016	−0.029	0.465	0.026	0.069	0.087	0.025
HbA1c	−0.077	0.046	0.063	0.016	0.678	0.016	−0.060	0.129	0.028	0.073	0.062	0.026
Triglycerides	−0.073	0.065	0.062	−0.087	0.031	0.023	0.002	0.963	0.025	0.136	<0.001	0.036
HDL cholesterol	0.050	0.207	0.060	0.046	0.256	0.018	0.047	0.250	0.027	−0.098	0.016	0.029
Aspartate aminotransferase	−0.066	0.100	0.062	−0.058	0.157	0.019	0.060	0.137	0.028	0.094	0.021	0.028
Alanine aminotransferase	−0.103	0.011	0.067	−0.101	0.014	0.025	0.100	0.014	0.033	0.104	0.011	0.030
Gamma-glutamyl transpeptidase	−0.052	0.199	0.060	−0.095	0.021	0.024	0.087	0.034	0.031	0.074	0.074	0.025
Fatty liver index	−0.105	0.012	0.066	−0.129	0.003	0.029	0.040	0.347	0.026	0.172	<0.001	0.043
LSM	−0.038	0.302	0.059	0.017	0.651	0.016	0.025	0.497	0.026	0.022	0.560	0.021

The multivariate analysis was adjusted for age, sex, smoking habits, exercise habits, and medication for hypertension, dyslipidemia, or diabetes mellitus. β, standardized coeffi-cient; R^2^, coefficient of determination; CAP, controlled attenuation parameter; BMI, body mass index; HbA1c, hemoglobin A1c; HDL, high-density lipoprotein; LSM, liver stiffness measure; MASLD, metabolic dysfunction-associated steatotic liver disease.

## Data Availability

The original contributions of this study are included in this article. Further inquiries can be directed to the corresponding authors.

## Abbreviations

The following abbreviations are used in this manuscript:

MASLD	metabolic dysfunction-associated steatotic liver disease
IL	interleukin
TNF	tumor necrosis factor-alpha
NAFLD	non-alcoholic fatty liver disease
NASH	non-alcoholic steatohepatitis
CAP	controlled attenuation parameter
SLD	steatotic liver disease
LDL	low-density lipoprotein
HDL	high-density lipoprotein
